# Spatially resolved and multiplexed MicroRNA quantification from tissue using nanoliter well arrays

**DOI:** 10.1038/s41378-020-0169-8

**Published:** 2020-05-09

**Authors:** Maxwell B. Nagarajan, Augusto M. Tentori, Wen Cai Zhang, Frank J. Slack, Patrick S. Doyle

**Affiliations:** 1 Department of Chemical Engineering, Massachusetts Institute of Technology, Cambridge, MA 02139 USA; 2 HMS Initiative for RNA Medicine, Department of Pathology, Harvard Medical School, Beth Israel Deaconess Medical Center, Boston, MA 02215 USA

**Keywords:** Engineering, Chemistry

## Abstract

Spatially resolved gene expression patterns are emerging as a key component of medical studies, including companion diagnostics, but technologies for quantification and multiplexing are limited. We present a method to perform spatially resolved and multiplexed microRNA (miRNA) measurements from formalin-fixed, paraffin-embedded (FFPE) tissue. Using nanoliter well arrays to pixelate the tissue section and photopatterned hydrogels to quantify miRNA, we identified differentially expressed miRNAs in tumors from a genetically engineered mouse model for non-small cell lung cancer (K-ras^LSL-G12D/+^; p53^fl/fl^). This technology could be used to quantify heterogeneities in tissue samples and lead to informed, biomarker-based diagnostics.

## Biomarkers: spatially resolved, multiple microRNA quantification from tissue

MicroRNA is a short, noncoding RNA molecule that regulates many biological processes and is emerging as a biomarker; a method has been developed to perform spatially resolved, measurements of multiple microRNA directly from formalin-fixed, paraffin-embedded (FFPE) tissue. Spatially resolved gene expression patterns are becoming a key area in medical studies, including diagnostics, but technologies for quantification and multiplexing in this area have been limited. Using a mouse model, a team headed by Patrick S. Doyle at the Massachusetts Institute of Technology, USA has succeeded in developing a method based on nanoliter well arrays for microRNA quantification. The team demonstrated that its technology allows greater multiplexing and better special resolution than can be achieved with existing methods. The authors consider that their technology has excellent potential for quantifying heterogeneities in FFPE tissue for next-generation biomarkers.

## Introduction

Spatially resolved and multiplexed measurements of biomolecules in tissue are emerging as key components of biological and medical studies, including basic research, pharmaceutical development, and companion diagnostics^1^. MicroRNAs (miRNAs) are small, noncoding RNAs that have been shown to be key regulators of multiple biological processes and are emerging biomarkers for many diseases^2^. Compared with mRNAs, miRNAs have higher stability in biofluids and tissues and higher tissue specificity^3,4^. MiRNA measurement may provide more information than mRNA measurement^5^ because of the transcriptional bursts associated with mRNAs^6,7^. Importantly, in formalin-fixed, paraffin-embedded (FFPE) tissue, the most common clinical sample storage type for patient tissue^8^, miRNAs are less likely to fragment than long RNAs, such as mRNAs, because of their smaller size^9^. Owing to the heterogeneity within tissues, methods such as miRNA in situ hybridization and fluorescence in situ hybridization^10^ (FISH) have demonstrated the clinical value of measuring the spatial layout of miRNAs, including in cancerous tissues^11^. For example, in non-small cell lung cancer tissue, high expression of miR-21 in tumor cell clusters predicted a favorable clinical outcome, whereas high expression of miR-21 in the stroma predicted a poor outcome^12^. The potential of miRNA FISH is limited, however, because it has not been used to measure more than two miRNAs from the same sample^13^. Panels of miRNAs have been shown to effectively classify cancers and assess tissue for disease state^5^. For example, the diagnostic potential for several diseases has been demonstrated for miRNA libraries containing three^14^, four^15^, five^16^, and seven^17^ different miRNAs measured simultaneously.

There are limited technologies that can measure miRNA with spatial resolution and multiplexing. Although combinatorial FISH-labeling techniques such as MERFISH^18^ can simultaneously image 100–1000 RNA species, in these techniques, the RNA strands need to be labeled with multiple probes, which is not possible with miRNAs because of their small size (~22 base pairs). Laser capture microdissection (LCM) followed by quantitative reverse transcription PCR (RT-PCR) is an approach that can increase the multiplexing of miRNAs with some spatial resolution, but this method generally requires specialized and expensive equipment and large tissue areas to capture sufficient RNA (100–1000 cells)^19,20^. Sequencing-based methods, such as fluorescent in situ sequencing^21^, spatial transcriptomics^22^, and Slide-seq^23^, have recently generated interest because they can achieve high levels of multiplexing and spatial resolution. However, spatial transcriptomics and Slide-seq capture mRNA using oligo dT primers, which are incompatible with miRNA since they have no poly(A) tail. Fluorescent in situ sequencing has not demonstrated the ability to measure miRNA, and it is not clear how to perform typical library preparation and small RNA enrichment steps as in typical miRNA sequencing protocols without disrupting the spatial information within the tissue. A technology that can measure miRNAs with spatial resolution and multiplexing directly from FFPE tissue could provide valuable information to researchers and lead to biomarker-based diagnostics.

## Results

**Fig. 1:**
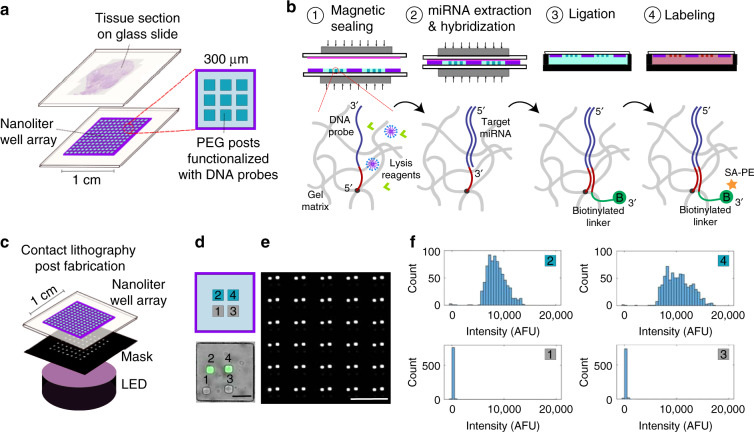
Tissue assay schematic and post fabrication. **a** Schematic of the nanoliter well array-tissue assay. **b** Steps of the miRNA tissue assay. 1, Magnets are used to seal the tissue section slide and array slide together. 2, Lysis reagents (sodium dodecyl sulfate and proteinase K) digest the tissue, and free miRNA hybridizes to DNA probes copolymerized within the hydrogel posts. 3, Arrays are placed in a buffer containing a biotinylated linker DNA sequence, which binds to DNA probes within the hydrogel and is ligated to the bound miRNA with DNA ligase. Biotinylated linkers that are not ligated to a miRNA are washed away. 4, Streptavidin-R-phycoerythrin (SA-PE), a fluorophore conjugated to streptavidin, is added and binds to the biotin on the linker sequences. Bound miRNA is quantified by measuring the mean fluorescence in a post using a slide scanner. **c** Schematic of the post fabrication process using contact lithography. **d** Four posts were polymerized in an array, where the first post was blank, the second post was biotinylated, the third was blank, and the fourth was biotinylated. SA-PE was added as in **b** step 4. Brightfield and green pseudocolored fluorescent channels were merged. Scale bar, 100 µm. **e** Fluorescent image of a subset of the full array from **d**. Scale bar, 500 µm. **f** Histograms of each post from the full array from **d**, **e**

Here, we present a method that can perform spatially resolved and multiplexed quantification of miRNA from FFPE tissue sections with no additional sample preparation. We combined a nanoliter well array with functional hydrogel (polyethylene glycol) posts to capture miRNAs and an FFPE tissue section mounted on a glass slide (Fig. 1a). Adapting a protocol from previous work^24,25^, we used magnets to compress the array and the tissue section together, whereas reagents (sodium dodecyl sulfate and proteinase K) within the nanoliter wells were used to digest the tissue and liberate the miRNA (Fig. 1b). Probes copolymerized within the hydrogels captured the miRNAs. A universal biotinylated linker sequence was then ligated to the miRNA with a templated ligation reaction, and streptavidin-R-phycoerythrin (SA-PE), a fluorophore conjugated to streptavidin, labeled each miRNA-binding site with a fluorophore. Although ligation approaches in library preparation for techniques such as miRNA sequencing can impart bias based on sequence^26–28^, our templated ligation reaction has been shown to be highly efficient (>95% in 10 min) because of the high ligation rate of T4 DNA ligase at the 3' end of the RNA to the 5' end of the DNA^29^. At these high ligation efficiencies, we anticipate negligible ligation bias based on sequence. Using our method, we pixelate the tissue sections (5 µm thick) into 300 µm pixels. Within each pixel, we can quantify up to nine different miRNAs using one fluorophore through spatial multiplexing. Unlike most methods for assaying FFPE tissue, our method does not require additional sample preparation steps because the hydrogels we use, made from polyethylene glycol diacrylate and treated with potassium permanganate, do not bind nonspecifically to components in FFPE tissue and can withstand the conditions needed to melt the paraffin, remove formaldehyde crosslinks, and digest the tissue^25^. Translating our previous work measuring miRNA in FFPE tissue from a 50 µL scale^25^ to a 3.5 nL scale achieves ~100× greater sensitivity^24^. By capturing miRNA on a different surface rather than directly labeling the tissue, we can incorporate negative controls into the assay.

### Device fabrication

To fabricate our devices, we first used a polydimethylsiloxane (PDMS) mold to mount a nanoliter well array on a glass slide, as described in previous work^24^. We simultaneously polymerized one hydrogel post in each well by aligning a chrome mask with the nanoliter well array over a UV light source^30^ (Fig. 1c and Fig. S1). Posts with different functionalities were polymerized by washing in between the polymerization steps and then aligning the mask to polymerize at a different point within the array. To assess the reproducibility of our fabrication method for multiplexing, we alternated polymerizing blank posts and functional posts containing a biotinylated probe (Fig. 1d–f, and Fig. S2). After incubation with SA-PE, we observed that 0.6% of the posts were lost in the process (10 out of 1568 fluorescent posts). These results demonstrate that we can polymerize posts with multiple functionalities throughout the 1 cm × 1 cm nanoliter well array used here.

### Multiplexed synthetic miRNA assay

We used these arrays to quantify miRNA by incorporating probes complementary to particular miRNAs within the posts. We polymerized nine posts within each well, each post containing probes complementary to a different miRNA. We built a calibration curve by adding synthetic miRNA targets to each of the wells and sealing against a glass slide with magnets (Fig. S3, Fig. S4, and Table S1). We found that the average limit of detection for the miRNAs tested here was 0.023 attomole, the same order of magnitude as the limit of detection demonstrated in previous work, 0.025 attomole^24^. Beyond prior work, we can simultaneously fabricate one post in each well, enabling 784 parallel, multiplexed assays from a tissue section.

### Spatially resolved and multiplexed miRNA tissue assay

To assess the reproducibility of our assay, we tested our nanoliter well arrays on serial FFPE tissue sections from a K-ras^LSL-G12D/+^; p53^fl/fl^ genetically engineered mouse model for non-small cell lung cancer^31^. We added lysis reagents (sodium dodecyl sulfate and proteinase K) to the wells to enable miRNA measurement directly from FFPE tissue, as described previously^25^ (Supplementary Note 1). To assess the reproducibility of our tissue assay, we measured miR-21 from serial sections of a tissue containing both tumor nodules and histologically normal adjacent to tumor tissue (NAT) (Fig. S5). Each well included a post targeting miR-21, a negative control post targeting cel-miR-54 (a miRNA from *Caenorhabditis elegans* not expected in this sample), and a negative control blank post. In each serial section, the heatmaps qualitatively matched the outline of the tissue sections tested, and the tumor tissue and NAT were significantly different (Fig. S5a–d). The coefficient of variation of miR-21 in region 1 for the four sections was 17%, which represents a combination of the assay and biological variability across these four sections. Because the thickness of these sections was 5 µm, cells present in one section may not be present in other sections tested, potentially leading to some biological variation. Using additional serial sections, we found that the assay signal increased up to an assay hybridization time of 3 h, with no additional improvements after 3 h (Fig. S6).

**Fig. 2:**
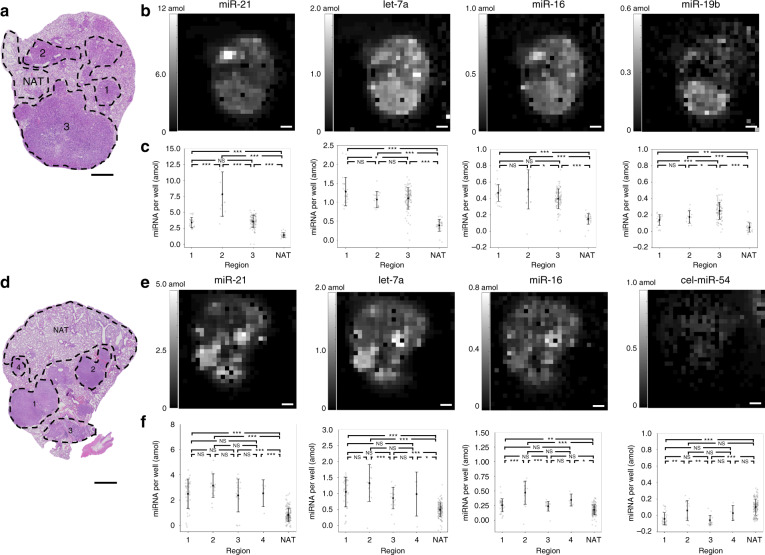
Multiplexed miRNA tissue assay finds differential miRNA expression in different tumors from the same mouse. **a**, **d** H&E staining of a proximal tissue section. Different regions are circled, with tumor regions labeled and normal adjacent to tumor tissue labeled NAT. Scale bars, 1 mm. **b**, **e** Heatmaps for four miRNAs measured during a multiplexed miRNA assay from this section. The brightness of each pixel in the heatmap corresponds to the amount of miRNA measured in one 300 µm × 300 µm well, where each well is separated by 50 µm. **c**, **f** Quantitative analysis of the different regions of the tissue. Error bars indicate one standard deviation. Gray dots indicate individual wells within the array. NS indicates not significant, *p < 0.05, **p < 0.01, ***p < 0.001 from Tukey’s honest significant difference test

We performed multiplexed miRNA assays on two tissue sections from different mice using nine-post arrays targeting eight different miRNAs (Fig. 2 and Fig. S7). Hematoxylin and eosin (H&E) staining of proximal tissue sections was used to label the tumor regions and the NAT (Fig. 2a, d). Heatmaps (Fig. 2b, e) and scatter plots (Fig. 2c, f) are shown for posts targeting several miRNAs, including cel-miR-54, a miRNA from *C. elegans* that was used as a negative control. The nanoliter well arrays could qualitatively capture the outline of these tissues, visually distinguishing between the tumor tissue and the NAT. We observed significant differences between the tumor tissue and the NAT in both sections, but interestingly, we also observed significant differences between the miRNA profiles for different tumor regions within the same mouse tissue section (Fig. 2a–c). For example, we observed that tumor region two expressed miR-21 at higher levels than tumor regions one and three, and we observed that tumor region three expressed miR-19b at higher levels than tumor regions one and two. These results are possibly due to distinct clones initiating these tumors. Our data showing increased expression levels of miR-21 and let-7a from NAT to tumor tissue are consistent with a recent study in lung cancer patients^32^. To assess differences in the vertical direction through the tumor, we also assayed a section proximal to that in Fig. 2a–c and a section ~100 µm away from the other sections in the same mouse FFPE block (Fig. S8). The three tissue sections showed similar trends in miRNA expression profiles, with the average coefficient of variation over the three sections for each miRNA in each region being 14% (Table S2).

### Comparison with RT-PCR

We compared the results from the section in Fig. 2a–c to the results from LCM quantitative RT-PCR. We dissected cells from each of the four regions from Fig. 2a, extracted RNA, and performed RT-PCR to measure the miRNA. The cells from two proximal sections were pooled to ensure that there was enough RNA for the assay. The LCM RT-PCR data were normalized against miR-26b, a commonly used endogenous control miRNA, and then normalized to NAT. MiR-26b was not detected by the nanoliter well array method, so for comparison, the nanoliter well array method was normalized by the approximate cell count and the NAT region (Fig. S9). As miR-26b is expected to be expressed by all cells at similar levels, we expect that normalizing by approximate cell count will lead to similar results. There are qualitative similarities in the trends for miR-21 and let-7a. However, the fold change measured by RT-PCR is much greater than that with the nanoliter well method. One explanation is that the NAT, which has fewer cells, is closer to the nanoliter well method’s limit of detection. Noise in the measurement may be causing the fold change to decrease below what was found with RT-PCR. One potential reason why the trends match for miR-21 and let-7a but do not appear to match for miR-16 is that RT-PCR and the nanoliter well array methods may have sequence biases that make them more likely to identify particular sequences than others. RT-PCR is known to have biases because of the amplification efficiencies of different sequences^33–35^. Thus, direct comparison between the two methods may be imperfect. Importantly, in this work, the nanoliter well method achieved better spatial resolution and used fewer sample preparation steps than LCM RT-PCR.

## Discussion

Here, we present a new method to quantify miRNA with multiplexing of up to nine and a spatial resolution of 300 µm. We demonstrated this technique using non-small cell lung cancer tissue from a genetically engineered mouse model, and we found significant differences between different tumor regions within the same mouse tissue section. Although miRNA FISH can measure miRNA with spatial resolution, the greatest multiplexing reported in the literature is 2-plex^13^. LCM RT-PCR can perform multiplexed measurements, but the typical spatial resolution is 100–1000 cells^20^, and PCR approaches can lead to bias in measurement^33–35^. For comparison, although the number of cells in a 300 µm × 300 µm square well would vary between tissue types and cell types, we counted ~80–160 cells per well in this work by counting nuclei in the H&E stain of a proximal section. This finding is consistent with our previous work, which demonstrated that a similar nanoliter well array operates effectively measuring ~10–100 cells^24^. Sequencing-based approaches can measure many RNAs that are much longer than miRNAs, but it is not clear how the library preparation and small RNA enrichment steps that typically occur in miRNA sequencing protocols can be used without disrupting the spatial position of the miRNAs within tissue. Specifically, spatial transcriptomics and Slide-seq capture mRNAs using oligo dT primers^22,23^. These are incompatible with typical mature miRNAs present in tissue because miRNAs lack poly(A) tails. Even if FISSEQ were compatible with miRNA, the typical FISSEQ protocol takes ~14 days to complete, making it impractical for many applications^36^. Multiplexed FISH techniques such as MERFISH are incompatible with miRNA because they require multiple probes per targeted sequence^21^. In vivo imaging techniques have demonstrated imaging of miRNAs in live cells and mice with multiplexing of up to two miRNAs^37,38^, but these technologies are suited for different, complementary applications. Our technology was designed to make measurements directly from FFPE tissue. Moreover, most other techniques require many processing steps before measuring miRNA from FFPE tissue.

One current limitation of our technology is that because our method measures miRNA from an isolated tissue area and does not use any target amplification, this approach cannot measure some miRNAs that are expressed at low levels in tissues. The tradeoff is that our method enables more robust quantitation of miRNA than other methods that involve target amplification, which can show target sequence bias. Future versions of our technique could use a signal amplification technique that remains localized in the gel. For example, in prior work using gel microparticles, we implemented rolling circle amplification to amplify the signal (not target), which led to an increase in sensitivity by over an order of magnitude^39^. Second, our method can achieve 9-plex assays, which is significantly greater than that of miRNA FISH^10^ and comparable to library sizes in existing miRNA diagnostic tests for miRNA measurement from bulk samples^14,17^. If greater levels of multiplexing are needed, we can increase the multiplexing by either making the posts smaller to fit more posts in each well or to use spectral multiplexing, which labels different miRNAs within the same post with different fluorophores. For example, 36 posts of 20-µm could fit in each well, and nonoverlapping fluorophores could measure 3–4 different miRNAs per post.

Prior work has modeled the kinetics of this miRNA assay. As the off-rate of the miRNA–probe interaction is much slower than the timescale for this assay^40^, the signal after the assay is governed by how much miRNA can diffuse to a probe and bind^24^. In our system, the reaction rate is much faster than the diffusion rate because of the high concentration of probes in the hydrogels^40^. Prior work found that 1.5 h of hybridization was much greater than the time for miRNA to diffuse through a well and bind to a probe^24^. In the tissue assays in this work, we found that hybridizing for 3 h gave a significantly higher signal than that for 1.5 h, and after 3 h, no significant additional improvements in signal were observed. It is likely that additional time is required to digest tissue and release free miRNA into the wells but that after 3 h, limited miRNA is released.

The well size and post size can be adapted for a particular application using this nanoliter well array approach. There is a balance between the spatial resolution and how much miRNA is present within the tissue. If we decrease the well size, there will be less miRNA present per well. Even if the concentration of miRNA in a tissue is approximately the same for different well sizes, because the off rate of the miRNA–probe interaction is much slower than the timescale of the assay^40^ and our hybridization step is much longer than the timescale for miRNA diffusion and reaction to a post^24^, the total amount of miRNA per well is relevant to the signal following the assay, not the concentration. Based on our previous experience with similar tissue sections, we expected ~5 attomole miR-21/mm^2^ tissue^25^. We chose 300 µm wells because we expected to be able to measure multiple miRNAs at this length scale and still be able to make multiple measurements of each tumor to assess heterogeneities within tumors (see Supplementary Note 2 for a calculation and comparison to previous work). The well shape and post shape can also be fine-tuned depending on the application. Previous work measuring miRNAs from cells used circle posts and wells^24^, whereas here, we used square posts and wells for better packing and easier registry across a tissue section. We can also reduce the well and post size to improve spatial resolution. Previous work has demonstrated photolithography of hydrogel features of 1.25 µm in size^41^ and fabrication of wells in arrays as small as 15 µm in diameter^42^. By implementing amplification schemes as described above, reducing well size, and reducing post size, we could work towards single cell resolution in future work.

There is growing interest in technologies to measure spatial gene expression patterns, and this nanoliter well array technology has demonstrated greater multiplexing and spatial resolution than existing methods. We envision that this approach could be a tool for researchers to study heterogeneities in FFPE tissue for next-generation biomarkers.

## Materials and methods

### Well array fabrication

Glass slides (24 mm × 60 mm × 0.16 mm) (VWR VistaVision Cover Glass, No. 1 ½) were acrylated as described previously^24^. Slides were submerged in a solution of two parts 3-(trimethoxysilyl)propyl methacrylate, three parts acetic acid, and five parts deionized water for 30 min at room temperature. The slides were rinsed with methanol and DI water before storing in vacuum. PDMS (Sylgard 184, Dow Corning) molds were made by mixing elastomer base and curing agent in a 10:1 ratio and cured at room temperature for at least 72 h on an SU-8 (MicroChem) master prepared using standard photolithography protocols. Features on the SU-8 master were measured using a profiler (Veeco, Dektak 150). The features were square wells with dimensions of 300 µm × 300 µm with a depth of 39 µm and spacing between wells of 50 µm. PDMS molds were removed, and 1.5 mm inlets were punched through the PDMS using a Biopsy Punch (Miltex). The PDMS molds were then placed on acrylated slides in a vacuum chamber for at least 1 h, the slides were removed from the vacuum, and Norland Optical Adhesive 81 (NOA81) was placed on the inlet to load the mold through degas-driven flow^43^. When the molds were filled (~15 min), they were cured under a UV lamp (Blak-Ray UV Bench Lamp, UVP) for 6 min. After curing, the molds were removed from the slides, and the slides were stored under vacuum until use.

### Hydrogel post fabrication

Slides with NOA81 arrays were degassed in DI water for 10 s in a benchtop sonicator (Branson 2800) to remove air bubbles in the wells. The devices were placed with the array facing down on 500 µm spacers made from three layers of electrical tape (3 M) attached to a glass plate (Corning Micro Slides, Plain, Pre-Cleaned 75 mm × 50 mm). Then, 1× Tris EDTA buffer with 0.05% Tween 20 (1× TET buffer) was pipetted between the array and the glass plate beneath.

Prepolymer solutions were prepared containing 18% (v/v) poly(ethylene glycol) diacrylate 700 g/mol, 36% (v/v) PEG 200 g/mol, 4.5% (v/v) Darocur 1173 photoinitiator (2-hydroxy-2-methylpropiophenone), ~1× Tris EDTA buffer (1 × TE), and DNA probes. Sequences and prepolymer concentrations are listed in Table S3. A blank prepolymer solution containing no DNA probe was also prepared. To load these solutions to a well array, the slide was removed from the spacers, excess buffer was removed from around the array by vacuum, and 20 µL of blank prepolymer solution was placed on the array. A pipette tip was used to spread the solution over the array for 1 min. Excess solution was removed by vacuum. This step was followed by three washes with 8 µL of prepolymer containing DNA probe, spread by a pipette tip for 30 s each. A slab of PDMS (~5 mm thick and large enough to cover the array) was placed on top of the array to seal it, and the slide was aligned and brought into contact with a chrome mask (FineLine Imaging) with 40 µm × 40 µm squares spaced such that one square fits within each well on our contact lithography setup^30^. The distance between the chrome mask and the prepolymer is the thickness of the slide, 0.16 mm. Posts were polymerized by sending UV light (1.5 mW cm^−2^, Thorlabs) through the mask and array for 300 ms. The PDMS cover was removed, and the washing steps with 20 µL of blank prepolymer and three washes with 8 µL of DNA probe prepolymer were repeated for each additional post. After all posts were polymerized, the devices were rinsed by flowing 1× TET over the array, placing the device face down on the spacer and washing three times in 1× TET (~1 mL total volume per array). The posts were treated with potassium permanganate (Sigma) to reduce nonspecific binding to the posts by continuously flowing a solution of 0.1 mol L^−1^ Tris-HCl buffer (pH 8.8) and 500 µmol L^−1^ potassium permanganate across the devices on tape spacers for 5 min. The devices were washed four times with 1× TET (~1 mL total volume per array). The devices were stored at 4 °C in 1× TET on tape spacers until use in an assay.

### miRNA assay

Adapted from previous work^24,25^, the miRNA assay consists of four steps: assembly, hybridization, labeling, and imaging. 1. Assembly: 1× TET with 350 mmol L^−1^ NaCl was loaded into the prepared arrays while they were still on the tape spacers to establish the salt conditions during hybridization. For synthetic miRNA assays, the hybridization buffer was 1× TET 350 mmol L^−1^ NaCl and synthetic miRNA (sequences shown in Table S3), and for tissue assays, the hybridization buffer was 1× TET with 350 mmol L^−1^ NaCl, 2% (w/v) sodium dodecyl sulfate (Sigma Aldrich), and 16 U mL^−1^ proteinase K (New England Biolabs). Arrays were loaded with hybridization buffer by removing them from the spacers and applying 25 µL of hybridization buffer to the array and spreading quickly with a pipette tip for 10 s. The arrays were sealed against either an FFPE tissue section mounted on a glass slide or a clean glass slide for the synthetic miRNA assay. A clean glass slide was placed on the opposite side of the array slide to support it. For the tissue assay, if an image of the array before the assay was desired, clamps (plastic, 1 inch jaw opening, McMaster Carr) were used to hold the two slides together to allow an image to be taken. Magnets were used to press the array and tissue or slide together continuously throughout the assay. 2. Hybridization: For the tissue assay, array-tissue sandwiches were heated at 55 °C for 15 min, 80 °C for 15 min, and then 55 °C for the remainder of the hybridization (either 1.5 h, 3 h, 6 h, or 16 h). During the heating steps, sodium dodecyl sulfate and proteinase K in the hybridization buffer lyse the tissue and liberate miRNA. For the synthetic miRNA assay, the hybridization step was at 55 °C for 1.5 h. Following hybridization, the arrays were removed and washed four times with rinse buffer (1× TET and 50 mmol L^−1^ NaCl) on the tape spacers for a total of approximately 1 mL of solution. 3. Labeling: A total of 250 µL of ligation solution containing 1× NEBuffer 2 (New England Biolabs), 1× TET, 800 U mL^−1^ T4 DNA ligase (New England Biolabs), 250 µmol L^−1^ ATP (New England Biolabs), and 40 nmol L^−1^ biotinylated linker (sequence is shown in Table S3) was pipetted under the array sitting on the spacers. The arrays were incubated in this solution for 1 h, protected from light, at room temperature. The arrays were then washed four times with rinse buffer (1 mL total volume) and incubated with 10 µg mL^−1^ SA-PE in rinse buffer for 1 h at room temperature, protected from light. After incubation, the arrays were washed again four times with rinse buffer as before and placed in rinse buffer for 1 h to allow excess SA-PE to diffuse out of the posts. 4. Imaging: Slides were removed from the spacers. An 18 mm × 18 mm coverslip was applied on top of the array and sealed with nail polish. The slides were glued to a 75 mm × 25 mm glass slide to be compatible with the GenePix 4400 A Slide Scanner (Molecular Devices). Slides were imaged with a 532 nm laser at full power, gain of 500, focal height of 100 µm, and spot size of 5 µm and with an Alexa 568 filter. Representative images of individual wells at higher magnification and in brightfield were taken using a Zeiss microscope. If arrays from the synthetic assays were reused, the arrays were placed on spacers in 1× TET and heated to 75 °C for 30 min and washed four times in 1× TET.

### Analysis

The slide scanner generated a tiff image that we used to quantify the signal from each post. For data corresponding to Fig. 2d–f, before analyzing the results, a preprocessing code was run to remove bright dust particles from the array. This code found connected pixel areas higher than a threshold value and larger than the post size and set these areas to background levels. Other arrays did not need this preprocessing step because of improvements in array handling. To generate a value for each post, we chose two wells and identified the pixel at the upper left corner of each post. Using this information, our code can find each post and calculate its mean intensity. For the synthetic miRNA assays, a window of wells with 9 rows × 11 columns was chosen from the center of the array to avoid edges and cracks if the array was cracked.

### Tissue-section preparation

All research involving mice complied with protocols approved by the Beth Israel Deaconess Medical Center (BIDMC) Biological Resource Center Institutional Animal Care and Use Committee (protocol 102–2014). The K-ras^LSL-G12D/+^; p53^fl/fl^ genetically engineered mouse model for non-small cell lung cancer was established at the BIDMC as reported previously^31^. Both male and female mice at 6 weeks of age were utilized for tumor initiation. K-ras^LSL-G12D/+^; p53^fl/fl^ mice have cre-dependent expression of oncogenic K-ras^G12D^ from the endogenous locus and p53 mutant variants with both p53 alleles deleted. Ad-cre (Gene Transfer Vector Core Facility at the University of Iowa) was delivered via intranasal inoculation. After euthanasia of a mouse, a whole lung was dissected from the mouse and prepared for histological analysis. Mouse lungs were formalin-fixed, paraffin-embedded, sectioned, and stained with H&E according to standard histopathological techniques. In brief, after fixation in 4% paraformaldehyde overnight, the tissues were dehydrated with ethanol and xylene. Then, the tissues were embedded into paraffin blocks. Sections (5 µm) of paraffin-embedded tissue were cut and placed onto glass microscope slides (Gold Seal). A thickness of 5 µm was chosen because this is a typical thickness for immunohistochemistry and H&E staining^44,45^. For H&E staining, the sections were stained with hematoxylin and eosin Y (Vector), dehydrated, and then covered with mounting medium (Vector). The images were taken under a microscope (Olympus).

### LCM

LCM was performed to select cells from the different regions identified by H&E staining of nearby sections. Microdissection was performed on sections stained with Arcturus HistoGene LCM Frozen Section Staining solution (Applied Biosystems cat. no. KIT0401). The sections were microdissected using an infrared laser with an Arcturus Laser Capture Microdissection System (Applied Biosciences) according to LCM procedures. LCM cells were pooled from multiple Arcturus CapSure Macro LCM Caps (Applied Biosystems, cat. no. LCM0211, lot no. 1809154). The caps were transferred and fitted onto 0.5 mL microcentrifuge tubes for RNA extraction. A total of 400–800 cells were collected in each region of the tissue sections, and up to two sections were used.

### RNA extraction

Total RNA was extracted from each tissue region microdissected by LCM using the AllPrep DNA/RNA FFPE Kit (Qiagen, cat. no. 80234, lot # 160018809) according to the manufacturer’s instructions. The microdissected tissue sections on each cap were incubated with Buffer PKD (Proteinase K digestion) and proteinase K at 56 °C for 15 min followed by complete cooling on ice for 3 min. The mixtures were centrifuged for 15 min at 20,000 × *g*, and the supernatant was transferred and incubated at 80 °C for 15 min followed by mixing with buffer RLT and ethanol. The mixture was transferred to an RNeasy MinElute spin column. After centrifugation, the column membrane was washed with Buffer FRN (FFPE RNA buffer) and incubated with DNase I and Buffer RDD (DNA Digest Buffer) for 15 min at room temperature. Buffer FRN was added into the MinElute spin column followed by a second wash with collected flow-through. Then, the column was washed twice with Buffer RPE followed by centrifugation at full speed for 5 min. Then, 14 µL of RNase-free water was added to the spin column membrane to elute the RNA.

### RT-PCR

A total of 10 ng of RNA for each sample was used as input for consecutive reactions including poly(A) tailing, ligation, reverse transcription, and miR-Amp reaction with a TaqMan Advanced miRNA cDNA synthesis kit (Applied Biosystems, cat. no. A28007). The miRNA levels were then assessed by TaqMan Advanced miRNA Assay with TaqMan Fast Advanced miRNA master mix (Applied Biosystems, cat. no. 4444557). Each PCR plate was run in an RT-PCR instrument (Roche LightCycler 480 System) according to the manufacturer’s instructions^46^. Three technical replicates were applied for each sample. The miRNA levels were assessed by TaqMan Fast Advanced MicroRNA Assays (Applied Biosystems). The TaqMan miRNA probes were as follows: hsa-miR-21-5 (mmu-miR-21a-5p, 477975_mir), hsa-let-7a-5p (mmu-let-7a-5p, 478575_mir), hsa-miR-16-5p (mmu-miR-16-5p, 477860_mir), hsa-miR-19b-1-5p (mmu-miR-19b-1-5p, 477962_mir), mmu-miR-210-5p (mmu481649_mir), hsa-miR-20a-5p (mmu-miR-20a-3p, 478586_mir), hsa-miR-15b-5p (mmu-miR-15b-5p, 478313_mir) and hsa-miR-26b-5p (mmu-miR-26b-5p, 478418_mir). hsa-miR-26b-5p was used as an endogenous control for analyses of miRNA expression^47^.

### Statistical analysis

All statistical comparisons between different tissue regions were performed with Tukey’s honest significant difference tests. The number of samples for each region was the number of wells beneath each region. In the calibration curves (Fig. S2), each data point corresponds to 99 separate wells in a 9 × 11 well window. Data points for individual wells were discarded if either the post for that miRNA was missing or if large fluorescent debris was detected on the post. Missing posts were identified by a highly negative signal of < −1000 AFU (because the cel-miR-54 negative control post had a much higher signal than the experimental post), and one post with debris was removed with Grubbs’s test. In Fig. 2a–c, three data points were removed for being below −1000 AFU; in Fig. S5 section III, one data point was removed for being below −1000 AFU; in Fig. S8g–i, one data point was removed by a one-sided Grubbs’s test at a 99.9% confidence level; Fig. S8a–c and Fig. S9c show the same data as in Fig. 2a–c and thus were treated similarly.

## Supplementary information

Supplementary Materials

## Data Availability

All data required to evaluate the conclusions in the paper are in the paper and the Supplementary Materials. Additional data are available from the corresponding author upon reasonable request.
