# Supplementary material for: Spatially resolved and multiplexed MicroRNA quantification from tissue using nanoliter well arrays
Source: Microsyst Nanoeng. 2020 May 9;6:51. doi: 10.1038/s41378-020-0169-8 (PMC7211184; doi:10.1038/s41378-020-0169-8)
Supplement: Supplementary file 1 — Supplementary Materials [file 41378_2020_169_MOESM1_ESM.pdf]

Supplementary Materials

Spatially-Resolved and Multiplexed MicroRNA Quantification from Tissue Using Nanoliter Well Arrays

Maxwell B. Nagarajan,[1] Augusto M. Tentori,[1] Wen Cai Zhang,[2] Frank J. Slack,[2] Patrick S. Doyle[1*]

[1]Department of Chemical Engineering, Massachusetts Institute of Technology, Cambridge, MA, USA 02139
[2]HMS Initiative for RNA Medicine, Department of Pathology, Harvard Medical School, Beth Israel Deaconess Medical Center, Boston, MA, USA 02215
*Corresponding author. Email: pdoyle@mit.edu

Contents

Fig. S1. Hydrogel post fabrication.
Fig. S2. Full array with posts fabricated with biotinylated DNA probe from Fig. 1e.
Fig. S3. Calibration curves for eight miRNAs tested in the panel for the tissue section from Fig. 2.
Fig. S4. Representative wells from the calibration curve in Fig. S3.
Fig. S5. Tissue assay from serial sections for miR-21.
Fig. S6. Tissue assay with serial sections and varying hybridization times.
Fig. S7. Supporting images for Fig. 2.
Fig. S8. Multiplex assay from different sections from the same tumor.
Fig. S9. Comparison of nanoliter well array data and RT-PCR data.
Table S1. Calibration curve slope, intercept, R^2, and limit of detection from Fig. S3.
Table S2. Coefficients of variation by miRNA and region for the data in Fig. S8
Table S3. Nucleic acid sequences used in this work.
Supplementary Note 1. Estimate of miRNA capture efficiency by comparison to previous work.
Supplementary Note 2. Comparison of miRNA measured to previous work.

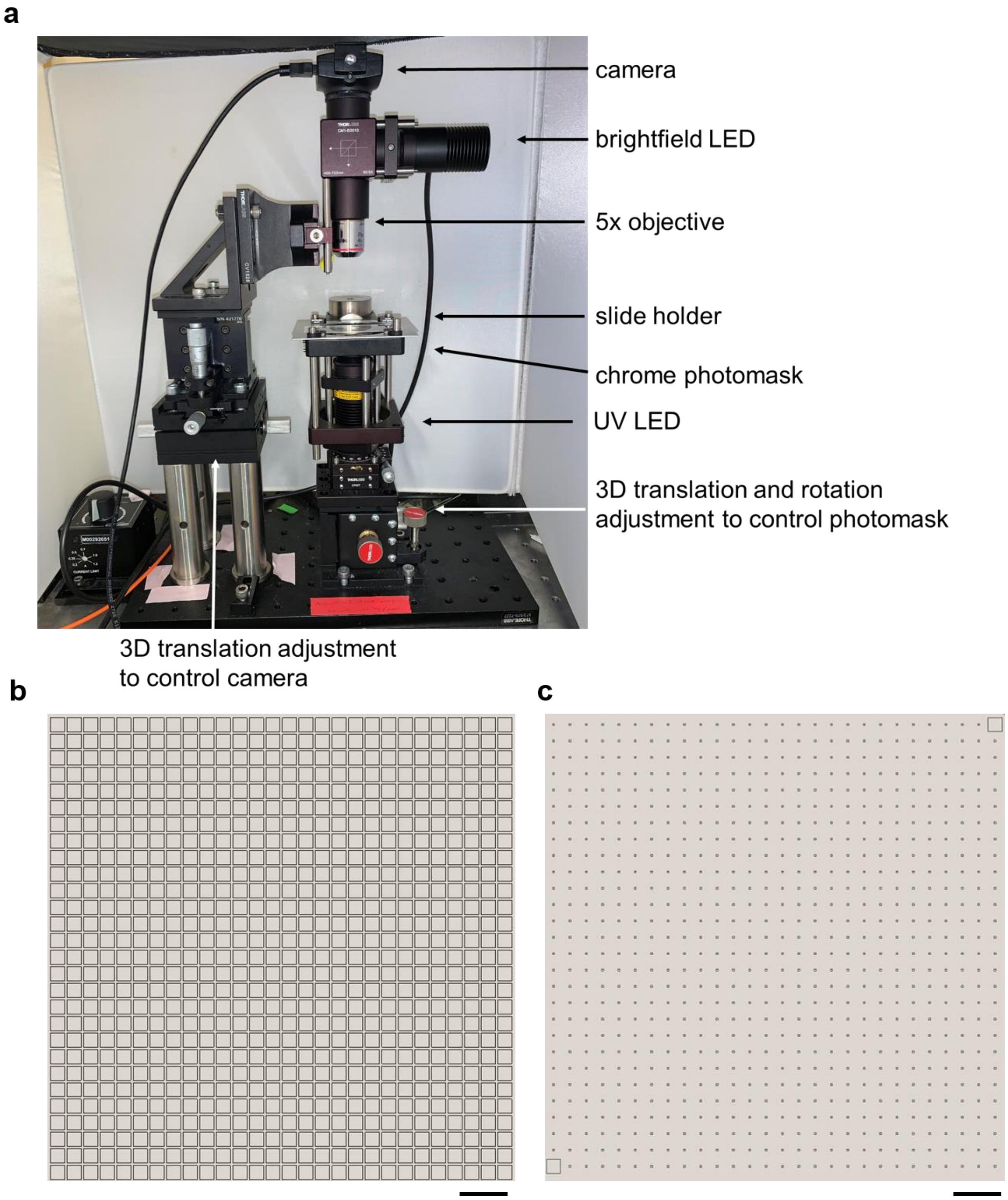

Fig. S1. Hydrogel post fabrication. (**a**) Contact lithography setup used to polymerize hydrogel posts. (**b**) Schematic of well array. Wells have 300 μm side lengths with 50 μm spacing between wells. (**c**) Schematic of photomask used to polymerize posts within the wells. Each square hole on the photomask has a 40 μm side length and 310 μm spacing. Holes on the photomask are spaced such that when aligned with the well array, each hole is in the same location of the corresponding well in the well array. Schematics were generated using AutoCAD. Scale bars 1 mm.

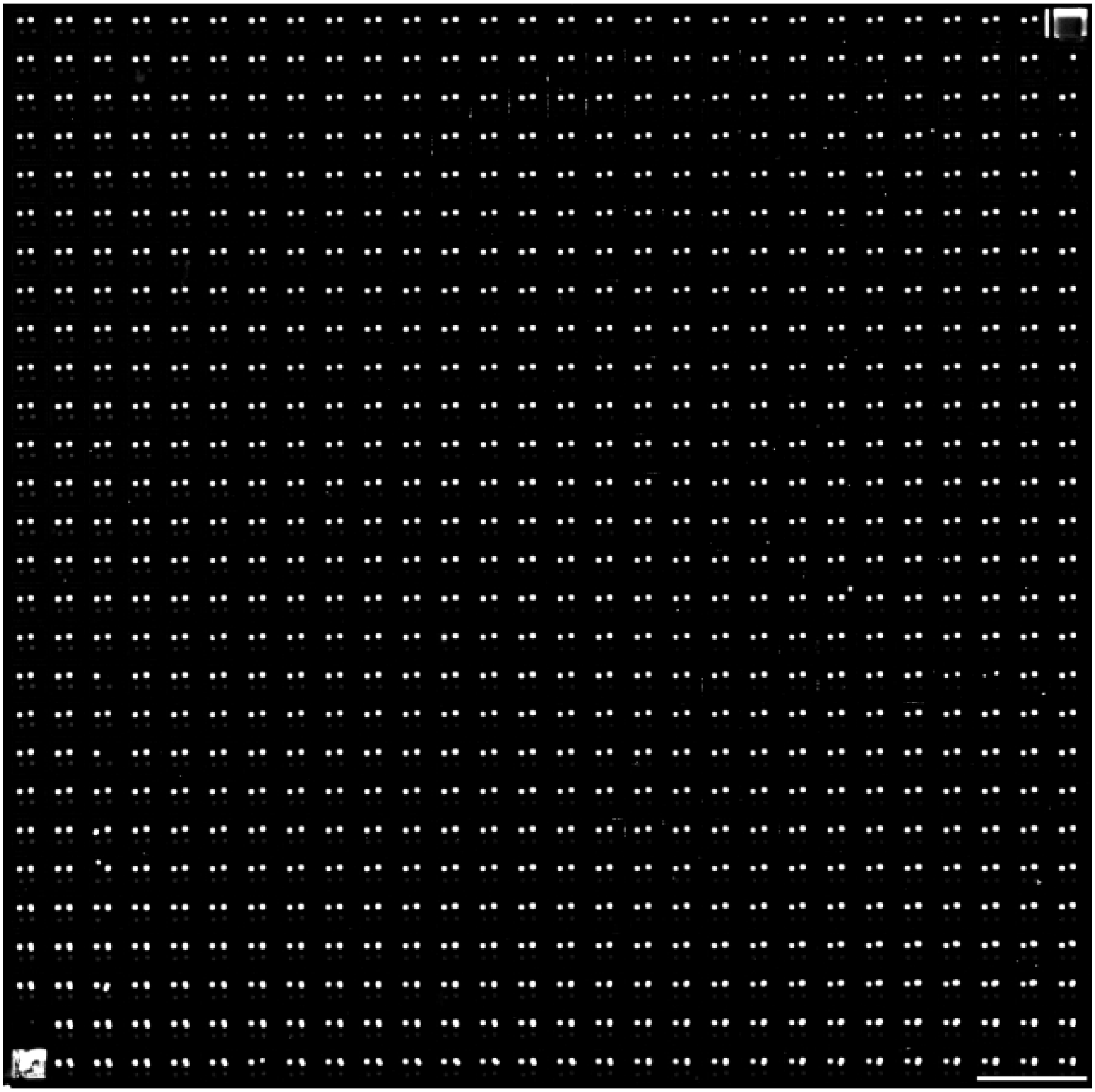

Fig. S2. Full array with posts fabricated with biotinylated DNA probe from Fig. 1e. As shown in Fig. 1d, four posts were polymerized, alternating blank and biotinylated. Streptavidin-R-phycoerythrin was added after fabrication to visualize probe incorporation in the functional posts and cross-talk between posts. Scale bar 1 mm.

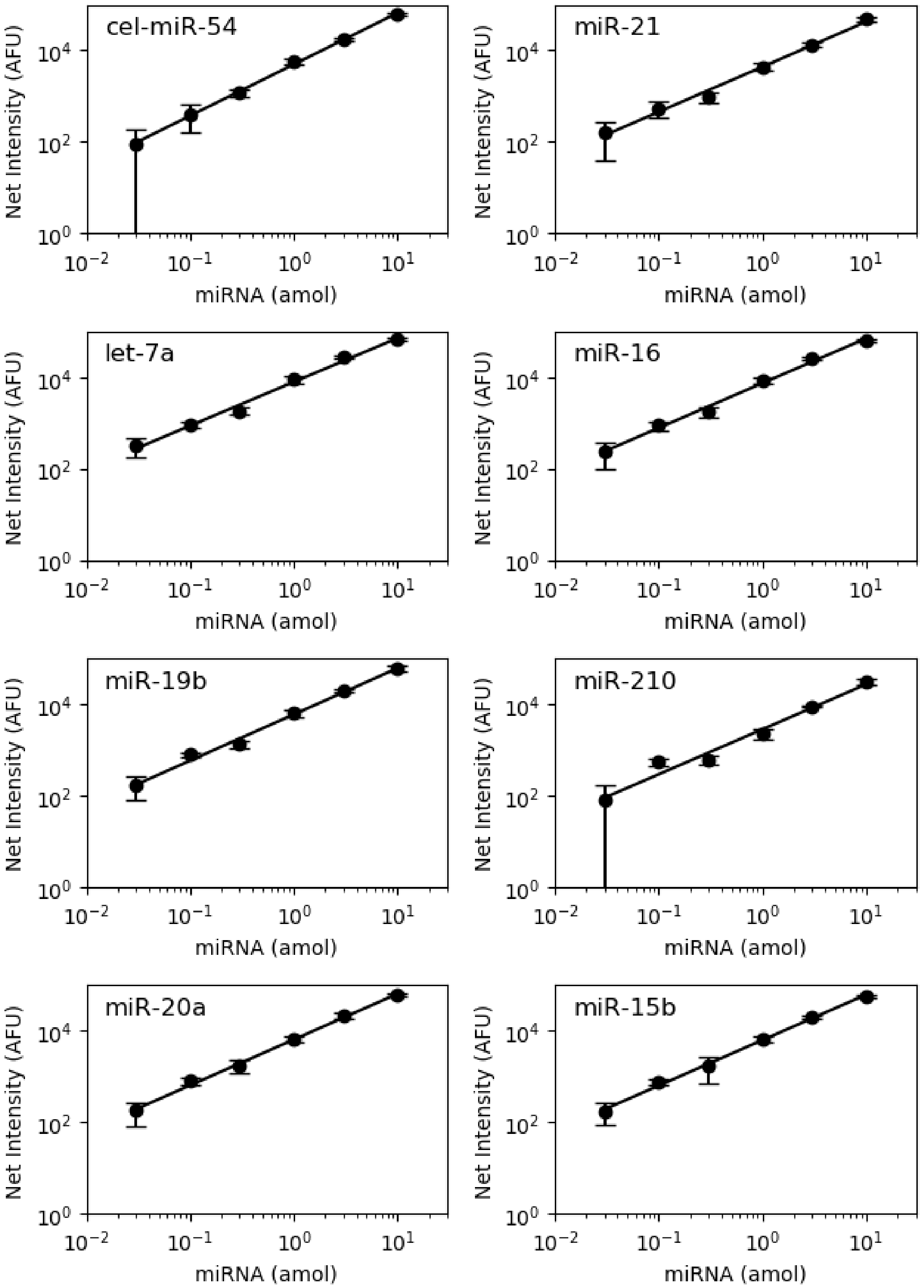

Fig. S3. Calibration curves for eight miRNAs tested in the panel for the tissue section from Fig. 2. Nine posts were polymerized in each device, one blank and eight with probes targeting the different miRNAs shown in the plots. Synthetic miRNA target was added to each array, and the arrays were sealed against a glass slide. Net intensity is the mean intensity of a post minus the mean intensity of the blank post. Representative wells are shown in fig. S4. Slope, intercept, and R^2 are shown in table S1.

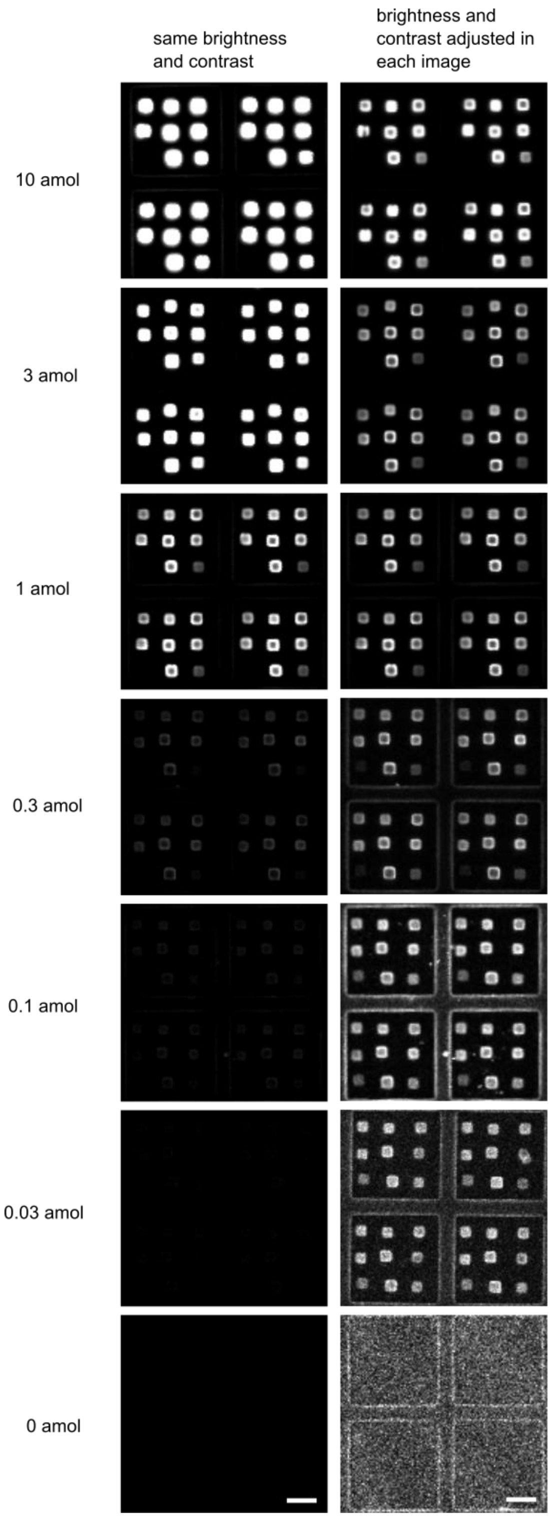

Fig. S4. Representative wells from the calibration curve in Fig. S3. The same images are shown on the left and right. In the left column, all images are set to the same brightness and contrast. In the right column, brightness and contrast are set individually. Eight posts targeting different miRNAs and one blank post (lower left corner of the well) were included. The same amount of synthetic miRNA was added for each post. Scale bar 100 µm.

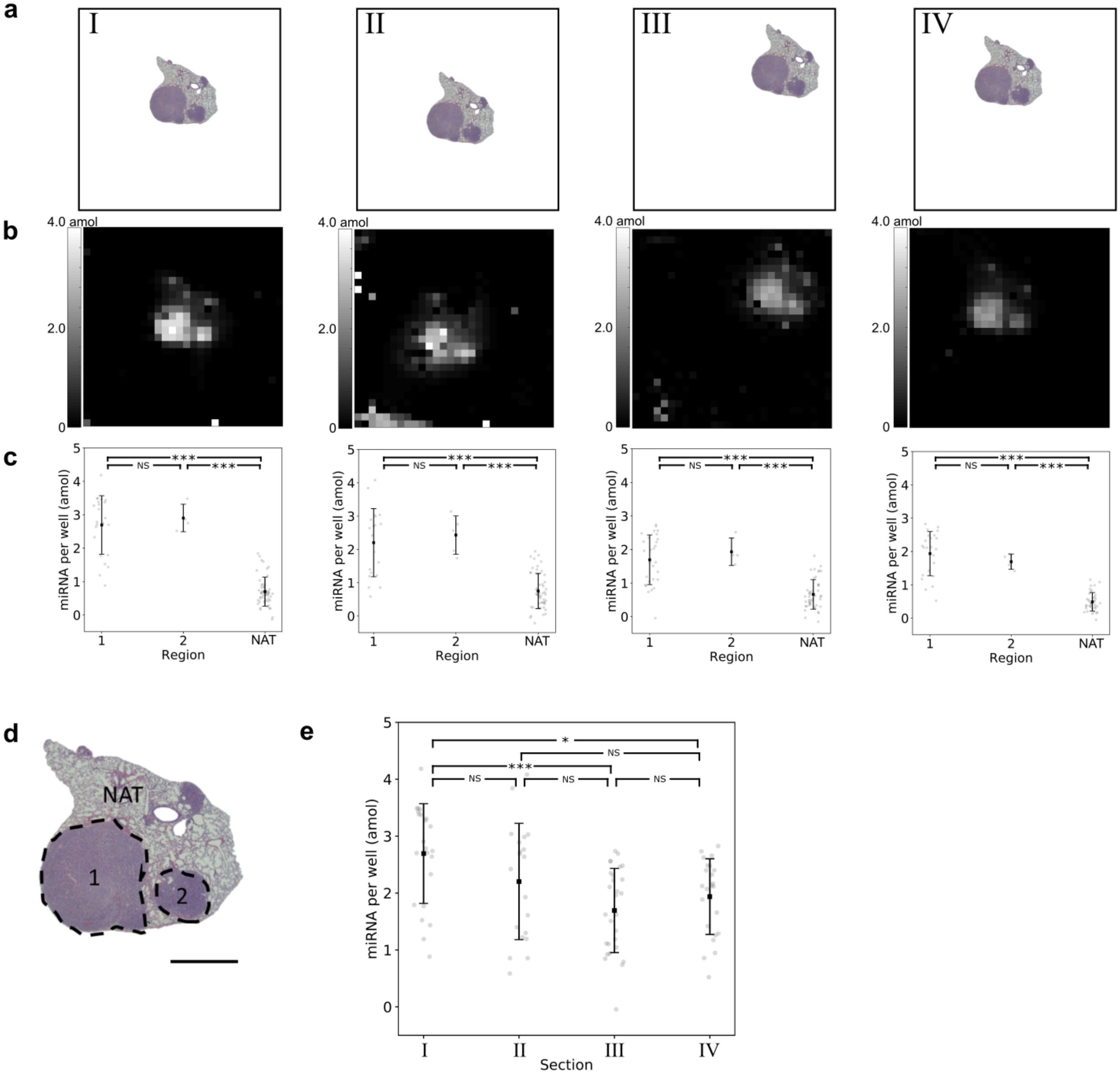

Fig. S5. Tissue assay from serial sections for miR-21. (**a**) Orientation of each of the four tissue sections with respect to the array, represented by the box. (**b**) Heatmaps for miR-21 of the four serial sections tested. The brightness of each pixel in the heatmap corresponds to the amount of miRNA measured in one 300 µm x 300 µm well, where each well is separated by 50 µm. (**c**) Quantitative scatterplots for each region of the tissue section (see part **d**). The black squares represent the means, the error bars represent one standard deviation, and the gray circles represent individual wells. (**d**) Diagrams of the regions of this tissue section on a nearby H&E stained section. NAT is normal adjacent to tumor tissue. Scale bar 1 mm. (**e**) Intensities for the four sections from region 1 for comparison between different serial sections. NS indicates not significant, * $p < 0.05$, ** $p < 0.01$, *** $p < 0.001$ from Tukey's honest significant different test.

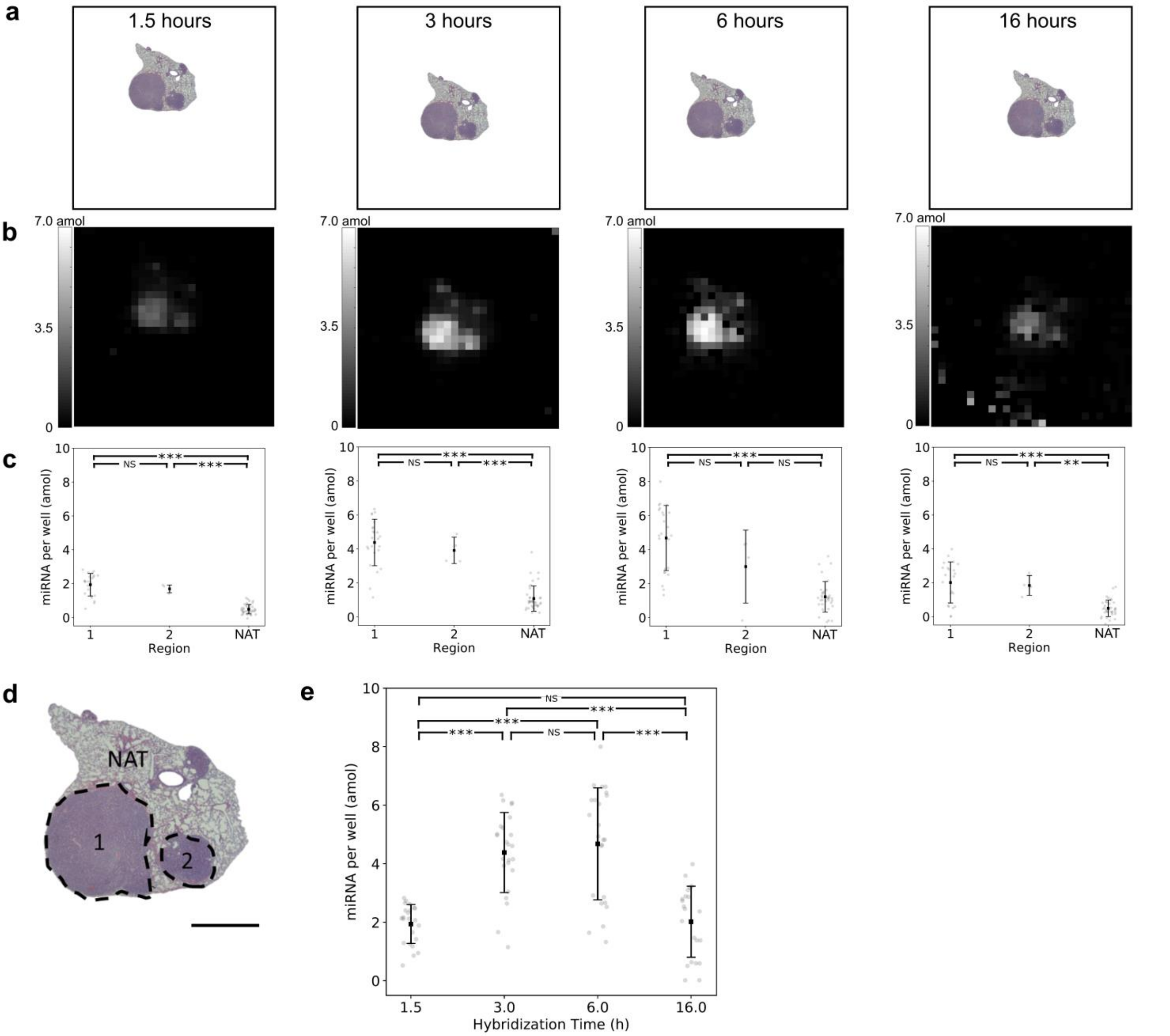

Fig. S6. Tissue assay with serial sections and varying hybridization times. Note that the 1.5 h section is the same as fourth section in Fig. S5 to best compare with later serial sections. (**a**) Orientation of each of the four tissue sections with respect to the array, represented by the box. (**b**) Heatmaps for miR-21 of the four serial sections tested. The brightness of each pixel in the heatmap corresponds to the amount of miRNA measured in one 300 µm x 300 µm well, where each well is separated by 50 µm. (**c**) Quantitative scatterplots for each region of the tissue section (see part **d**). The black squares represent the means, the error bars represent one standard deviation, and the gray circles represent individual wells. (**d**) Diagrams of the regions of this tissue section on a nearby H&E stained section. NAT is normal adjacent to tumor tissue. Scale bar 1 mm. (**e**) Intensities for the four sections from region 1 for comparison between different serial sections at different hybridization times. The 16-hour array dried out during the hybridization step, which is likely why this array shows a lower signal. NS indicates not significant, * $p < 0.05$, ** $p < 0.01$, *** $p < 0.001$ from Tukey's honest significant different test.

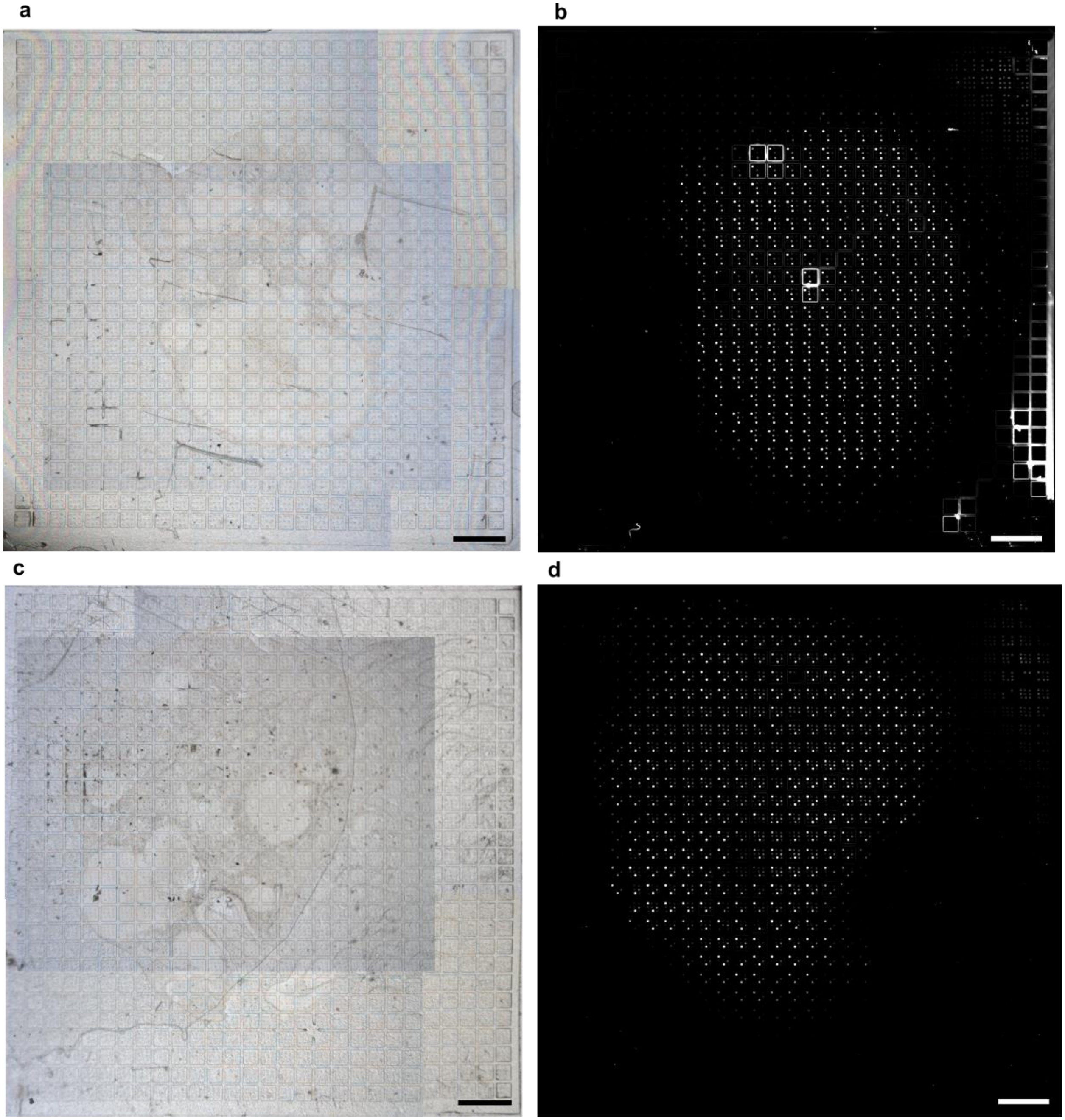

Fig. S7. Supporting images for Fig. 2. (**a**) Image taken before the assay from Fig. 2a-c, with array and FFPE tissue section together. (**b**) Fluorescent image of the array after the assay from Fig. 2a-c. (**c**) Image taken before the assay from Fig. 2d-f, with array and FFPE tissue section together. (**d**) Fluorescent image of the array after the assay from Fig. 2d-f. Scale bars 1 mm.

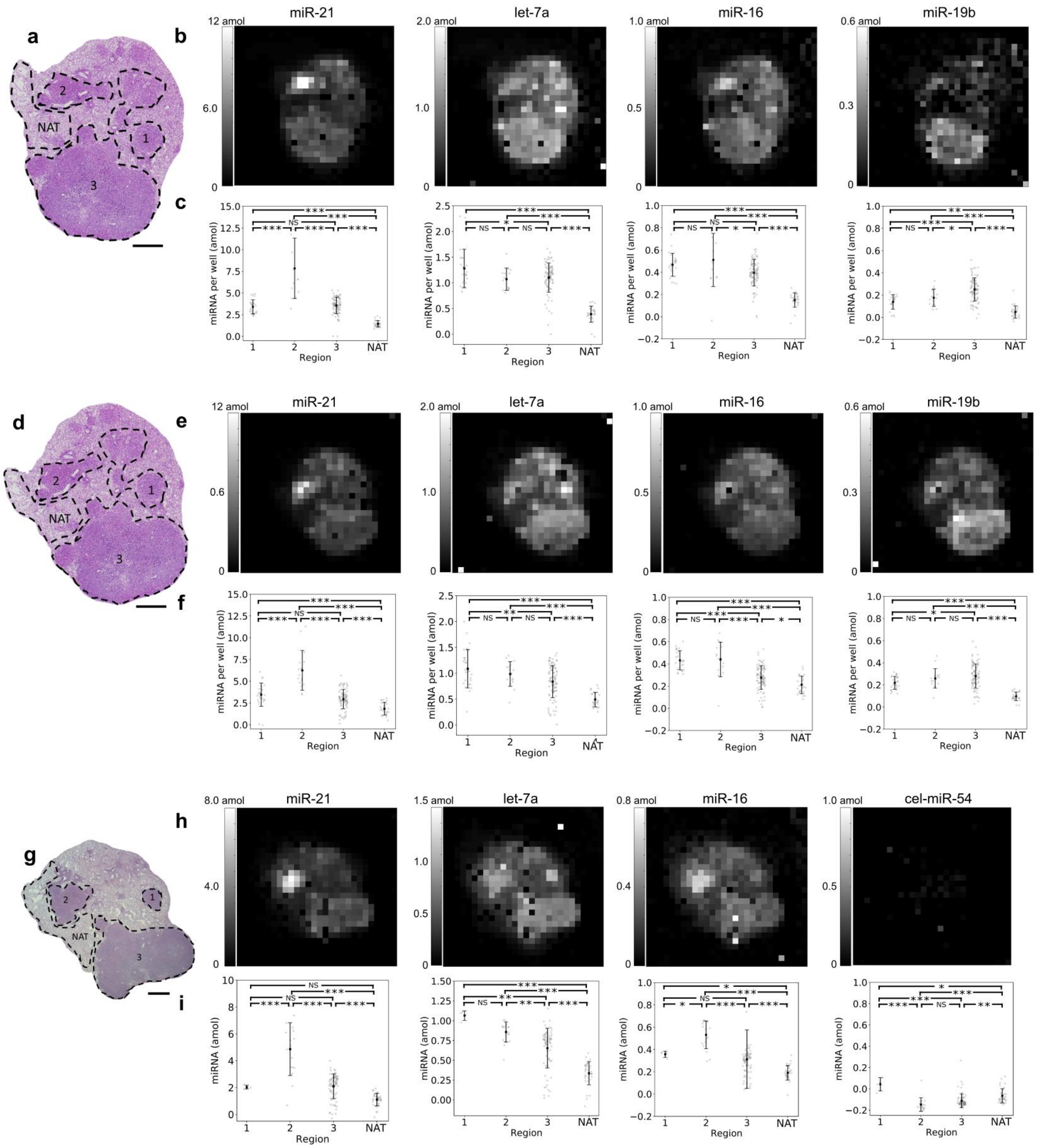

Fig. S8. Multiplex assay from different sections from the same tumor. (**a-c**) Same data as in Fig. 2, A to C. (**d-f**) Proximal section to that in **a-c**. (**g-i**) Section from the same tumor as **a-f**, but about 100 µm away in the z direction. (**a, d**, and **g**) H&E stain of a proximal tissue section. Different regions are circled, with regions 1, 2, and 3 tumors, and NAT is normal adjacent to tumor tissue. Scale bars 1 mm. (**b, e**, and **h**) Heatmaps for four miRNAs measured during a multiplex miRNA assay from this section. The brightness of each pixel in the heatmap corresponds to the amount of miRNA measured in one 300 µm x 300 µm well, where each well is separated by 50 µm. (**c, f**, and **i**) Quantitative analysis of the different regions of the tissue. Black squares represent the means. Error bars indicate one standard deviation. Gray dots indicate individual wells within the array. Negative values occur if the test post has less signal than the control post. NS indicates not significant, * $p < 0.05$, ** $p < 0.01$, *** $p < 0.001$ from Tukey's honest significant different test.

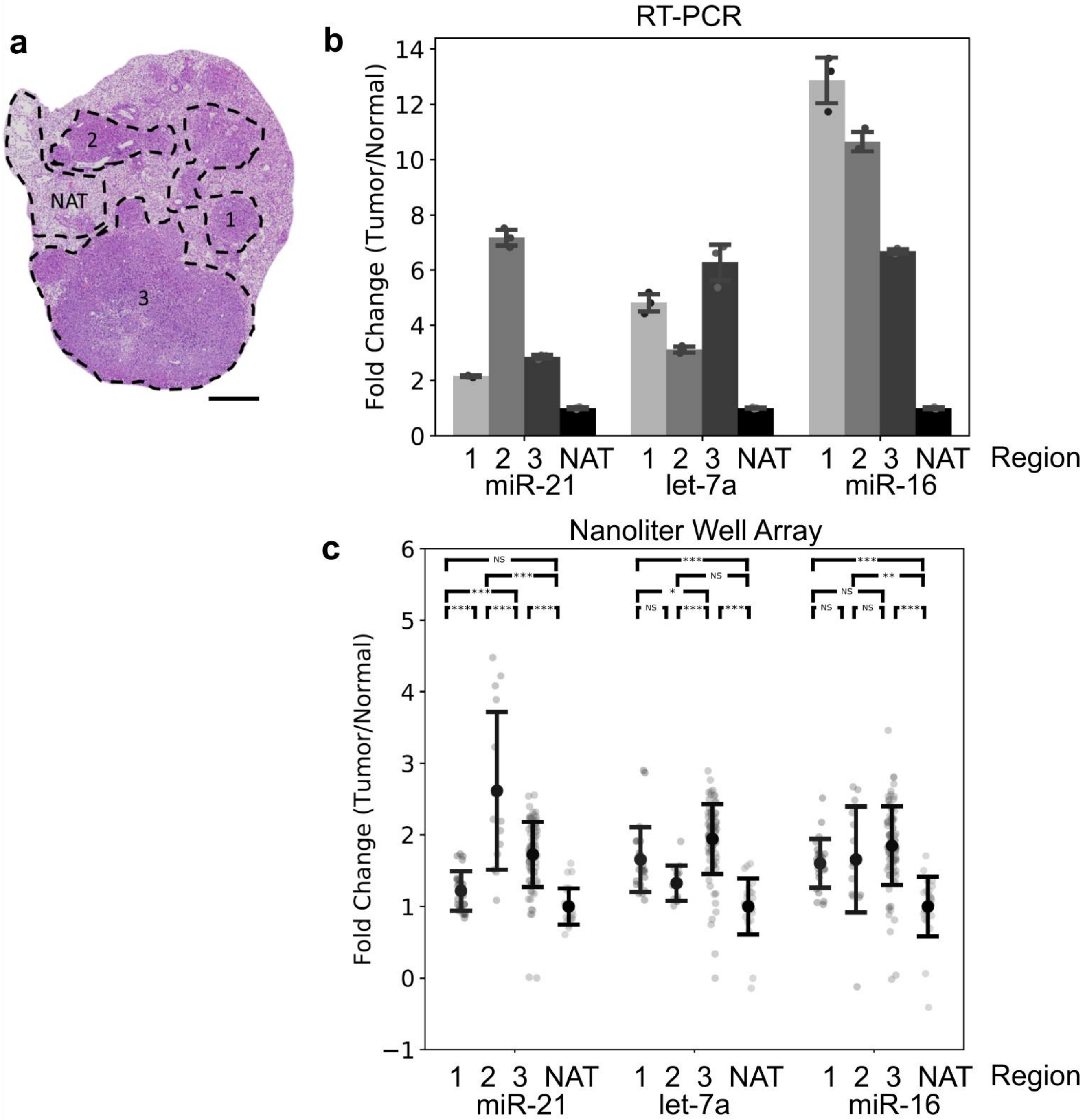

Fig. S9. Comparison of nanoliter well array data and RT-PCR data. (**a**) H&E stain of proximal tissue section showing the different regions. 1, 2, and 3 are tumors, and NAT is normal adjacent to tumor tissue. Scale bar 1 mm. (**b**) RT-PCR data normalized to endogenous control miR-26b, and then normalized to NAT. Error bars represent standard deviation. (**c**) Nanoliter well array data from Fig. 2a-c, normalized by cell number, and then normalized to NAT. Error bars indicate one standard deviation. Circles in **b** represent technical replicates. Gray circles in **c** indicate individual wells within the array. Trends in miRNA expression patterns agree between the two methods for miR-21 and let-7a. The fold changes are larger when using RT-PCR compared to the nanoliter well array method. NS indicates not significant, * $p < 0.05$, ** $p < 0.01$, *** $p < 0.001$ from Tukey's honest significant different test.

Table S1. Calibration curve slope, intercept, R^2, and limit of detection from Fig. S3. The natural log of the data was taken before calculating these values to account for the large range of values. Slope and intercept values in this table apply to the equation *ln*(signal) = slope × *ln*(miRNA) + intercept, where signal has units (AFU) from the slide scanner raw data output, and miRNA has units of amol.

Target	Slope	Intercept	R^2	Limit of Detection (amol)
cel-miR-54	1.13	8.52	0.999	0.028
miR-21	0.98	8.40	0.996	0.021
let-7a	0.96	8.99	0.996	0.0081
miR-16	0.98	8.93	0.997	0.013
miR-19b	1.01	8.70	0.996	0.016
miR-210	0.98	7.96	0.987	0.053
miR-20a	1.00	8.78	0.998	0.044
miR-15b	0.99	8.75	0.998	0.023

Table S2. Coefficients of variation by miRNA and region for the data in Fig. S8. Each element of the table is the standard deviation divided by mean of the three measurements in Fig. S8 for a particular miRNA in a particular region. The average of all elements of the table is 0.14.

	miR-21	let-7a	miR-16
region 1	0.190	0.073	0.097
region 2	0.153	0.079	0.075
region 3	0.167	0.162	0.124
Normal adjacent tissue	0.203	0.160	0.167

Table S3. Nucleic acid sequences used in this work. All DNA probes have a 5' Acrydite modification and a 3' inverted dT modification. The biotinylated linker has a 5' phosphate modification and a 3' biotin modification. Concentration listed is the concentration in the prepolymer solution if applicable. Concentrations of miR-21 and let-7a probe were adjusted to approximately rate match different probe sequences in prior work (ref. 29 in the main text). RNA sequences were used to generate calibration curves. All sequences were purchased from Integrated DNA Technologies.

**Name**	**Sequence**	**Concentration (µmol/L)**
DNA Sequences		
cel-miR-54-3p probe	GAT ATA TTT TAC TCG GAT TAT GAA GAT TAC GGG TA	100
miR-21-5p probe	GAT ATA TTT TAT CAA CAT CAG TCT GAT AAG CTA	247
let-7a-5p probe	GAT ATA TTT TAA ACT ATA CAA CCT ACT ACC TCA	50
miR16-5p probe	GAT ATA TTT TAC GCC AAT ATT TAC GTG CTG CTA	100
miR-19b-3p probe	GAT ATA TTT TAT CAG TTT TGC ATG GAT TTG CAC A	100
miR-210-3p probe	GAT ATA TTT TAT CAG CCG CTG TCA CAC GCA CAG	100
miR-20a-5p probe	GAT ATA TTT TAC TAC CTG CAC TAT AAG CAC TTT A	100
miR-15b-5p probe	GAT ATA TTT TAT GTA AAC CAT GAT GTG CTG CTA	100
miR-26b-5p probe	GAT ATA TTT TAA CCT ATC CTG AAT TAC TTG AA	100
biotinylate probe	ATA GCA GAT CAG CAG CCA GA	10
biotinylated linker	TAA AAT ATA TAA AAA AAA AAA A	
RNA Sequences		
cel-miR-54-3p	uacccguaaucuucauaauccgag	
miR-21-5p	uagcuuaucagacugauguuga	
let-7a-5p	ugagguaguagguuguauaguu	
miR16-5p	uagcagcacguaaauauuggcg	
miR-19b-3p	ugugcaaauccaugcaaaacuga	
miR-210-3p	cugugcgugugacagcggcuga	
miR-20a-5p	uaaagugcuuauagugcagguag	
miR-15b-5p	uagcagcacaucaugguuuaca	
miR-26b-5p	uucaaguaauucaggauaggu	

Supplementary Note 1. Estimate of miRNA capture efficiency by comparison to previous work.

Because there is no gold standard method for knowing the absolute amount of miRNA in any tissue section, we can only estimate the miRNA capture efficiency. In prior work using the same hydrogel assay chemistry from formalin-fixed, paraffin-embedded tissue, we evaluated the miRNA assay signal after sequentially considreing first formaldehyde crosslinks and second paraffin in the assay (Nagarajan et al. Anal Chem 2018). First, we found quantitative agreement between formalin-fixed cells and frozen cells, indicating the miRNA capture efficiency was not changed in the presence of formaldehyde crosslinks. This is likely because of the high stability of miRNA in formalin-fixed samples. Moreover, the copy number per cell in prior work measuring directly from cells is in agreement with expectations from the literature (Lee et al. Anal Chem 2016). Second, we found that when comparing miRNA assay signal in an FFPE tissue section and a proximal FFPE tissue section with paraffin removed by a xylene wash, the signal decreased by 10% when paraffin was included in the assay. This 10% reduction of signal could be caused by the presence of paraffin in the hybridization step of the assay reducing overall assay performance or by more difficult extraction of miRNA from paraffin-embedded tissue. Third, we performed a spike-in experiment in which we added synthetic cel-miR-54 (a miRNA not expected to be found in this tissue), to a tube containing FFPE tissue. We found that the signal on hydrogels targeting cel-miR-54 was 15% less in the tube containing FFPE tissue compared to a tube with no tissue. This experiment indicates the degree to which FFPE tissue may interfere with the assay. Based on these experiments, we estimate that the capture efficiency is between 75-85%, depending on if these two effects are additive or not.

Nagarajan, Maxwell B., et al. "Nonfouling, Encoded Hydrogel Microparticles for Multiplex MicroRNA Profiling Directly from Formalin-Fixed, Paraffin-Embedded Tissue." *Analytical Chemistry* 90.17 (2018): 10279-10285.

Lee, Hyewon, et al. "Encoded hydrogel microparticles for sensitive and multiplex microRNA detection directly from raw cell lysates." *Analytical Chemistry* 88.6 (2016): 3075-3081.

Supplementary Note 2. Comparison of miRNA measured to previous work.

We can also quantitatively compare the amount of miRNA measured in tissue from this work to prior work. In our prior work, using tissue sections from the same mouse model (K-ras$^{LSL\text{-}G12D/+}$; p53$^{fl/fl}$) for non-small cell lung cancer, but different mice, we found approximately 5 amol miR-21 / mm^2 of 5 µm thick FFPE tumor tissue (Nagarajan et al. Anal Chem 2018). In one 300 µm x 300 µm well in this work, we would expect

(5 amol miR-21 / mm^2) x (0.3 mm x 0.3 mm) = 0.45 amol of miR-21 per well.

When designing this study, we anticipated that this amount of miRNA per well would enable measurement of several miRNAs directly from tissue. In this work in Fig. 2, S5 S6, and S8, we observe typical miR-21 per well levels of between 2 amol and 5 amol. One potential cause of the five to ten-fold higher signal than expected is that these are tumors from different mice, and we have observed significant differential expression of miR-21 across tumors from even the same tissue section, as shown in Fig. 2a-c. Another potential reason for this difference is that a longer lysis and hybridization time would have captured more miRNA in previous work. This is illustrated by Fig. S6, where we see increases of signal up to three hours, whereas Nagarajan et al. Anal Chem 2018 used a ninety-minute hybridization time and a larger reaction volume. A smaller reaction volume improves the reaction kinetics (Pregibon et al. Anal Chem 2009), so even at the same hybridization time, more miRNA would be captured in the nanoliter well than in a larger 50 µL tube.

Nagarajan, Maxwell B., et al. "Nonfouling, Encoded Hydrogel Microparticles for Multiplex MicroRNA Profiling Directly from Formalin-Fixed, Paraffin-Embedded Tissue." *Analytical Chemistry* 90.17 (2018): 10279-10285.

Pregibon, Daniel C., and Patrick S. Doyle. "Optimization of encoded hydrogel particles for nucleic acid quantification." *Analytical Chemistry* 81.12 (2009): 4873-4881.

Supplementary Note 3. Post saturation in assays in this work.

We do not expect probes to become saturated with miRNA at these probe concentrations and amounts of miRNA within tissue. The concentration of DNA probe within the hydrogel posts is the concentration in the prepolymer (Table S3) multiplied by the probe incorporation, which we assume is approximately 11% as was found in previous work (Pregibon and Doyle *Anal Chem* 2009). Applying this calculation to miR-21, the miRNA with the greatest abundance within the tissue we used here, there is about 1.7 fmol of probe in each post. The highest amount of miR-21 measured in this work was about 12.5 amol miR-21 per well (Fig. 2c, region 2). Almost all other measurements, particularly for other miRNAs, were well below 10 amol. Thus, in the most extreme case of miR-21, the number of probe molecules in each post is more than two orders of magnitude higher than the number of free miRNA per well. In addition, we can apply the calculation to let-7a, the lowest concentration probe in Table S3. We estimate 340 amol let-7a probe per post, and we measure about 1 amol let-7a per well in our tissue assays. Similarly, the amount of probe per post for let-7a is more than two orders of magnitude higher than the amount of miRNA measured per well.

We also expect that the labeling steps provide excess SA-PE and enough time for SA-PE to fully diffuse through the gel and out of the gel. First, in Fig. 1d-f we observe that the posts with biotinylated DNA are about uniformly fluorescent. If SA-PE were not in excess or if additional time were required for SA-PE to diffuse through the gel, we would observe a boundary layer on these posts because SA-PE would be more likely to bind at the surface of the gel. Second, we observe a linear relationship between signal and amount of synthetic miRNA added to the wells in our calibration curves (Fig. S3 and Table S1). This is further justification that there is sufficient SA-PE added to our arrays up to at least 10 amol of miRNA per post per well. Moreover, note that more fluorophores are required in the highest calibration curve data point, where 10 amol of each miRNA was added to each well, than in any of the tissue assays, where less total miRNA per well bound to the posts. Thus, if SA-PE was in excess in the 10 amol data point in the calibration curve, it was in excess for each of the tissue assays reported here. Third, we confirm that there is sufficient time for SA-PE to diffuse out of the posts after the labeling step because the negative control posts in the calibration curve are difficult to distinguish from background (see Fig. S4 for an example well).

Pregibon, Daniel C., and Patrick S. Doyle. "Optimization of encoded hydrogel particles for nucleic acid quantification." *Analytical Chemistry* 81.12 (2009): 4873-4881.
